# Ten-year fatal and non-fatal myocardial infarction incidence in elderly populations in Spain: the EPICARDIAN cohort study

**DOI:** 10.1186/1471-2458-9-360

**Published:** 2009-09-24

**Authors:** Rafael Gabriel, Margarita Alonso, Blanca Reviriego, Javier Muñiz, Saturio Vega, Isidro López, Blanca Novella, Carmen Suárez, Francisco Rodríguez-Salvanés

**Affiliations:** 1 Unidad de Investigación. Red RECAVA. Hospital Universitario La Paz. Madrid, Spain; 2 Instituto Universitario de Ciencias de la Salud. Hospital Juan Canalejo. Red RECAVA. Universidad de La Coruña, Spain; 3 Centro de Salud Arévalo. Red RECAVA. Avila, Spain; 4 Unidad de Hipertensión. Red RECAVA. Hospital Universitario de la Princesa. Madrid, Spain

## Abstract

**Background:**

In Spain, more than 85% of coronary heart disease deaths occur in adults older than 65 years. However, coronary heart disease incidence and mortality in the Spanish elderly have been poorly described. The aim of this study is to estimate the ten-year incidence and mortality rates of myocardial infarction in a population-based large cohort of Spanish elders.

**Methods:**

A population-based cohort of 3729 people older than 64 years old, free of previous myocardial infarction, was established in 1995 in three geographical areas of Spain. Any case of fatal and non-fatal myocardial infarction was investigated until December 2004 using the "cold pursuit method", previously used and validated by the the WHO-MONICA project.

**Results:**

Men showed a significantly (p < 0.001) higher cumulative incidence of myocardial infarction (7.2%; 95%CI: 5.94-8.54) than women (3.8%; 95%CI: 3.06-4.74). Although cumulative incidence increased with age (p < 0.05), gender-differences tended to narrow. Adjusted incidence rates were higher in men (957 per 100 000 person-years) than in women (546 per 100 000 person-years) (p < 0.001) and increased with age (p < 0.001). The increase was progressive in women but not in men. Adjusted mortality rates were also higher in men than in women (p < 0.001), being three times higher in the age group of ≥ 85 years old than in the age group of 65-74 years old (p < 0.001).

**Conclusion:**

Incidence of fatal and non-fatal myocardial infarction is high in the Spanish elderly population. Men show higher rates than women, but gender differences diminish with age.

## Background

Coronary heart disease (CHD) is the main cause of death in the industrialized countries[1], and myocardial infarction (MI) the most dominant manifestation of the disease. In Spain, 11.2% of deaths in males and 9.6% in females were related to CHD in 2005, and more than 85% of annual CHD deaths occur in subjects older than 65 years[2] Although CHD mortality has declined steadily both in men and women[3], it still remains as one of the most frequent causes of mortality[4]

Incidence studies conducted in Spain indicate that CHD is a frequent disease in the general population, with rates in order of 200 new myocardial infarctions per 100,000 men-year and 50 per 100,000 women-year respectively[5] The higher incidence can be observed in the eldest age group. In Spain more than 58% of hospital discharges with the diagnosis of cardiovascular disease are over 65 years and 63% of MI patients admitted to hospitals are over this age[6]. Although higher rates are observed over the age of 65[2], most epidemiological studies still focus on the middle-aged adult population, with scarce representation of the elderly[7]. In fact, age for inclusion used to be limited to 65 years[8] and few studies include subjects older than 74 years[9]. The impact of striking demographic changes in the Spanish elderly population, with an increase of ten million elders during the last decade, and its effect on CHD incidence and mortality needs to be evaluated.

The aim of this paper is to estimate the age- and sex- specific incidence and mortality rates of MI in a cohort of Spanish elders during 10 years of follow-up (1995-2004).

## Methods

The EPICARDIAN project is a multicenter, population-based, epidemiological cohort study about cardiovascular diseases in the elderly population of three Spanish areas: Lista district (Madrid city); Arévalo county (Avila); and Begonte county (Lugo). In the urban district of Lista (Madrid city), where the number of registered elders was large (>25.000), we selected an age- and sex- stratified random sample of the population. In the other two study sites (Lugo and Arévalo), all registered elders were invited to the baseline examination. The response rate of the initial cohort selection was 85%. Study design and methods have been previously published[10,11]

### Population and design of the study

**Figure 1:**
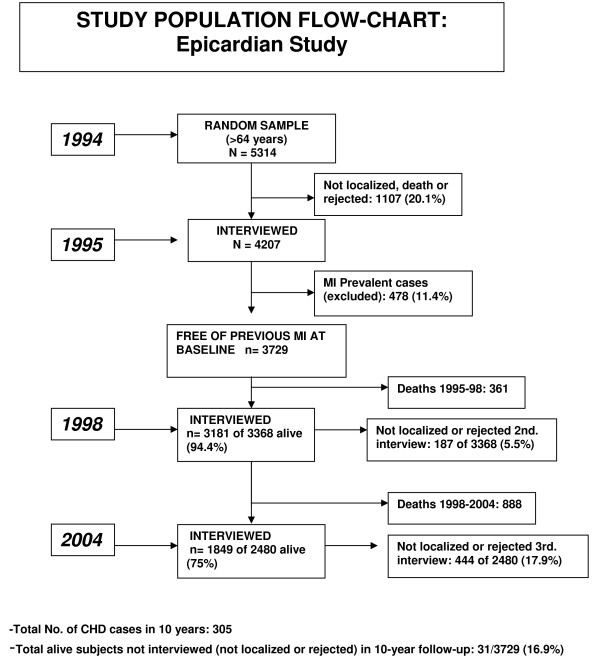
**Flow-chart EPICARDIAN study (1995-2004)**. Coronary heart disease (CHD).

The original cohort was composed of 3729 subjects older than 65 years old free of previous MI. All participants were followed until the occurrence of the first MI, until death or until 31/12/2004 if alive. Study design, response rates and follow-up information are shown in figure 1.

### Field study procedures

The fieldwork was developed into two phases. First phase included a home standard examination, consisting of an interview, physical examination and laboratory tests, in which information on newly developed cardiovascular disease was obtained. Registered nurses were trained and certified in performing the interview. The interview included questionnaires adapted and validated in Spanish from the MONICA project[12] and the Rose questionnaire on chest pain and dysnea[13]. In 1998 and 2004 this standard examination was repeated using the same contact procedures and methods than in the baseline (1995).

The second phase pertained to the confirmation of CHD events in those individuals who answered affirmatively the questions about coronary disease or angina in the first phase. We used the so called "cold pursuit method" previously used and validated by the MONICA Project, and modified and adapted in Spain by the REGICOR and IBERICA projects[14,15]. In the "cold pursuit method" cases are identified and registered through their discharge. We modified it for pin-pointing and investigating incident cases, employing all of the hospital and primary care clinical records for confirming the cardiovascular event. Causes and dates of death of the deceased individuals in the cohort were also confirmed through the Spanish National Death Index.

Cardiovascular information was requested for all potential lost cases using secondary sources (clinic history, interviewing assigned general practitioner, National Institute of Statistics, mails, and phone calls to the subject or relatives).

### Definition of myocardial infarction (MI)

Criterion was based on symptoms, enzyme changes, ECG changes (Minnesota code)[16,17] and post-mortem findings defined in the WHO coding criteria[18] and the validity of the diagnosis was ensured by intra-observer studies[19]

We considered *Definite MI *in those subjects who presented a definite ECG or typical/atypical symptoms, together with a probable ECG and abnormal enzymes, or those with typical symptoms, abnormal enzymes and ischemic ECG.

Possible MI or Coronary Death was considered in those alive subjects with typical symptoms where enzymes and ECG did not allow classification into the definite category, with no evidence of other diagnosis; or death patients with no clear evidence of other cause of death. A Coronary event was considered fatal if death occurred within

28 days of the onset. Under the epigraph "*sudden death*" were included those subjects who died within the first 24 hours from the onset of clinical symptoms, deaths without witness or when the subject was well in the previous 24 hours.

*No MI *was set in alive patients when the symptoms and diagnostic tests combinations did not allow its classification into the possible MI or when the event could be explained by means of other causes. In fatal events, MI was rejected when death was related to different clinical diagnosis or autopsy.

Ischaemic cardiac event includes all coronary events that satisfy the criteria for non-fatal definite myocardial infarction or coronary death (fatal definite infarction, fatal possible infarction, or sudden death).

### Statistical methods

Basic statistics of centralization (mean, median and its standard deviation and distribution values) were used to describe continuous variables. For the calculation of rates and proportions, the distribution of relative frequencies with confidence intervals at 95% by the Poisson approximated method were used.

To compare proportions the Pearson chi squared test or the chi squared for linear trend for several categories were used. Any value p < 0.05 was considered statistically significant. The Kaplan-Meier actuarial method was used in order to estimate the cohort survival and crude incidence rates. Crude incidence rates were standardized for age at baseline by the direct method, using as reference the European population distribution in 2006 as reference[20]

Cumulative incidence is calculated by the number of new cases during the period of study divided by the number of subjects at risk in the population at the beginning of the study. Mortality is the number of deaths in the population. Incidence rate is the number of first fatal and/or non-fatal events per unit of person-time at risk. Mortality rate is the number of fatal events per unit of person-time at risk. Case fatality is the proportion of fatal MI in all MI. Incidence and mortality rates are presented per 100,000 person-years, and case-fatality as a percentage by age at baseline and sex groups for the ten-year follow-up.

Our research is in compliance with the Helsinki Declaration . All participants signed a informed consent at the beginning of the study. The study protocol was approved by the ethics committee of the Hospital Universitario de la Princesa, Madrid.

## Results

General characteristics of the initial cohort (n = 3729) and distribution of major cardiovascular risk factors are described in table 1. The initial response rate was 85%. There were 1297 (34.8%) subjects from Lista, 989 (26.5%) from Begonte, and 1443 (38.7%) from Arévalo. The mean age of the cohort at baseline was 74.2 ± 0.4 years (74.43 in women and 73.82 in men). The initial cohort of 3729 subjects accounted for a total 27661 persons-year of follow-up. Median follow-up for the entire cohort was 7.4 years.

**Table 1:** Demographic characteristics and distribution of major cardiovascular risk factors in the EPICARDIAN study population at baseline (year 1995).

**Cardiovascular risk factors**		**Men** **n (%)** **n = 1592**	**Women** **n (%)** **n = 2137**	**Both** **n (%)** **n = 3729**
**Age group**	**65-74**	975 (61.2)	1228 (57.4)	2203 (59.0)
	**75-84**	487 (30.6)	692 (32.4)	1179 (31.7)
	**> = 85**	130 (8.2)	217 (10.2)	347 (9.3)
**Hypertension**		969 (60,8)	1497 (70,0)	2466 (66,1)
**Hypercholesterolemia**		347 (21,8)	709 (33,2)	1056 (28,3)
**Diabetes**		170 (10,7)	254(11,9)	424 (11,4)
**Obesity**		302 (18,9)	605 (28,3)	907 (24,3)
**Smoking**		340 (21,4)	69 (3,2)	409 (10,9)

Number of cases and their corresponding percentages (%)

Hypertension: SBP = 140 mmHg or DBP = 90 mmHg or antihypertensive treatment.

Diabetes: venous glucose ≥ 126 mg/dl or antidiabetic treatment.

Hypercholesterolemia: Cholesterol ≥ 250 mg/dL or lipid lowering treatment

Obesity: Body Mass Index (BMI) ≥ 30 kg/m^2^.

Smoking: 1 cigarette per day or 5 per week during the last year

In total, 305 CHD events occurred in the entire cohort during the 10-year follow-up period. There were 95 cases of non-fatal definite MI; 101 of fatal definite MI; 51 of possible MI, and 58 of sudden death (table 2). Men showed a higher and significant cumulative incidence of ischemic events (p < 0.001); definite MI (p < 0.001); fatal (p < 0.05) and non-fatal MI (p < 0.05); and sudden death (p < 0.05) than women. When we analyzed cumulative incidence by age we observed an increased incidence of fatal MI with age (p < 0.001) but incidence of non-fatal definite MI did not show a significant increase. The analysis by age, considering the age at the beginning of the study, also showed that men had higher non-fatal cardiovascular risk at earlier ages whereas women described a progressive increase in all cardiovascular risk.

**Table 2:** Cumulative number of cases and risk by sex and age of different CHD diagnostic categories in 10 years of follow-up (1995-2004).

**CHD diagnostic** **Categories**		**Males**				**Females**				**Total** **(95%CI)**	**p**
	
		**65-74**	**75-84**	**<85**	**All males** **(95%CI)**	**65-74**	**75-84**	**<85**	**All females** **(95%CI)**		
Non-fatal definite AMI	No. of events	38	15	3	56	19	14	6	39	95	

	%	3.9	3.1	2.3	3.5 (2.6-4.5) p = 0.53	1.5	2.0	2.8	1.8 (1.3-2.5) p = 0.42	2.5 (2.1-3.1)	<0.05

Possible AMI	No. of events	15	8	2	25	10	12	4	26	51	

	%	1.5	1.6	1.5	1.6 (1.0-2.3) p = 0.8	0.8	1.7	1.8	1.2 (0.8-1.7) p = 0.142	1.4 (1.0-1.8)	0.05

Sudden death	No. of events	13	14	6	33	2	19	4	25	58	

	%	1.3	2.9	4.6	2.1(1.4-2.9) p < 0.05	0.2	2.7	1.8	1.2 (0.7-1.7) p < 0.001	1.6 (1.2-2.0)	<0.001

Fatal and non-fatal definite AMI	No. of events	71	31	12	114	32	32	18	82	196	

	%	7.3	6.4	9.2	7.2(6.0-8.5) p = 0.51	2.6	4.6	8.3	3.8(3.0-4.7) p < 0.001	5.3 (4.6-6.0)	<0.001

Ischemic event	No. of events	99	53	20	172	44	63	26	133	305	

	%	10.2	10.9	15.4	10.8(9.3-12.3) p = 0.2	3.6	9.1	12	6.2 (5.2-7.3) p < 0.001	8.2 (7.3-9.1)	<0.001

Possible and non- fatal definite AMI	No. of events	53	23	5	81	29	26	10	65	146	

	%	5.4	4.7	3.8	5.1(4.0-6.2) p = 0.67	2.4	3.8	4.6	3.0 p = 0.085	3.9 (3.3-4.6)	0.01

*CI: Confidence Intervals. "p" represents differences by age and gender*. *Ischemic event *includes all coronary events that

satisfy the criteria for non-fatal definite myocardial infarction or coronary death (fatal definite infarction,

fatal possible infarction, or sudden death)

**Figure 2:**
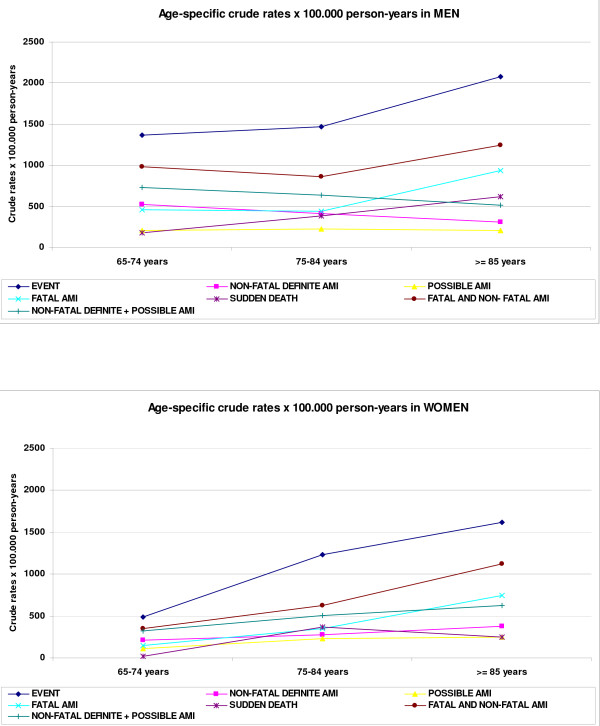
**Crude rates by sex and age per 100.000 person-years of different acute myocardial infarction diagnostic categories**. *Ischaemic event *definition includes all coronary events that satisfy the criteria for non-fatal definite myocardial infarction or coronary death (fatal definite infarction, fatal possible infarction, or sudden death).

Figure 2 graphically depicts sex and age-related differences in incidence rates (first event) of different acute myocardial infarction diagnostic categories. Rates progressively increase in women but not in men. Men showed similar incidence rates at 65-74 and 75-84 age-groups. Males and females older than 85 years showed the highest incidence rates. Age- standardized incidence rate (per 100 000 person-years) for first definite (fatal and non fatal) MI was greater in men (957; 95%CI: 899-1019) than in women (546; 95%CI: 514-606) (p < 0.001).

Among the 3729 subjects, 1249 died for any reason during the 10 years of follow-up, which represents a total mortality proportion of 33.5%. The cumulative mortality for CHD, MI or sudden death, was 4.3% (95%CI: 3.6-5.0), higher in men 5.7% (95%CI: 4.6-7.0) than in women 3.2% (95%CI: 2.5-4.0) (p < 0.001); (Table 2). Finally, MI as definite cause of death was confirmed in 58 subjects, which resulted in a specific MI mortality of 2.7% (95%CI: 2.2-3.3). MI mortality rate was also higher in males than in women and particularly among the eldest groups (p < 0.001). Case fatality also increases with age in both genders (p < 0.05) and was higher in men than in women (p < 0.01) (table 3).

**Table 3:** Myocardial infarction case fatality by age at baseline and sex among participants. EPICARDIAN (1995-2004)

**Gender**	**Age**	**Case fatality** **(95%CI)**	**No. of events** **Fatal MI/all MI**
**Men**	**65-74**	38% (29-49)	33/86
	**75-84**	41% (27-57)	16/39
	**>85**	64% (38-86)	9-14
**Women**	**65-74**	31% (18-46)	13/42
	**75-84**	41% (27-55)	18/44
	**>85**	55% (33-74)	12-22

**Total**	**>65**	41% (35-47)	101/247

Values represent proportion with fatal events within each group

All MI (Myocardial Infarctions): definite and possible

p for trends (age) <0.05; p (gender) <0.01

Crude CHD mortality rate was 575 per 100 000 person years with higher figures in men (770 per 100 000 person-years) than in women (428 per 100 000 person-years) (p < 0.001). MI mortality rate was 365 (95%CI: 329-404) per 100,000 person- years, greater in men (491; 95%CI: 449-536) than in women (271; 95%CI: 240-305) (p < 0.001). MI adjusted-mortality rates (table 4) were also higher in men (484; 95%CI 442-529) than in women (293; 95%CI 260-329) (p < 0.001). Population aged ≥ 85 years old at baseline reached the highest value with 815 cases per 100.000 person-years, twice compared to 75-84 years old (388 per 100.000 person-years), and three times compared to 65-74 years old (281 per 100.000 person-years) (p < 0.001).

**Table 4:** Incidence rates and mortality rates for acute myocardial infarction (AMI) in different national and international studies carried out in the elderly.

		**All incidence rates**		**All mortality rates**	
**Study** **Period of Study** **(Reference No.)**	**Age** **Group**	**Men**	**Women**	**Men**	**Women**
**EPICARDIAN Study** REGICOR-65 plus (24)*	**65-74**	981(921-1044) 599(528-670)	351(316-389) 166(131-202)	456(416-499) 220(177-263)	142(120-167) 55(35-75)
	**75-84**	858(802-917) 972(840-1105)	623(576-673) 409(338-481)	442(402-485) 570(468-672)	350(315-388) 223(170-276)
	**>85**	1244(1177-1314) 1071(791-1351)	1118(1054-185) 671(307-836)	933(875-994) 740(506-973)	745(693-800) 426(295-558)
EPICARDIAN Study** 1994-2004	**>65**	957(898-1018)	546(502-594)	484(442-529)	293(260-329)
**Oxford Study** **1994-95 (30)**	**65-79**	1585	868	826	478
**Rotterdam Study** **1990-2000 (26)**	**>55**	840(660-1020)1 420(280-550)2	310(230-390)1 360(2.60-450)2		
**Bronx Aging Study** **1981-89 (31)**	**>75**	4700 (total)		3900 (total)	
**Cardiovascular Health** **Study 1989-90 (32)**	**>65**	2070	790		
**Framingham Heart** **Study1960-1999 (33)**	**65-74**	1800	800		
	**75-84**	2700	1400		

**Age- standardization by the European Standard Population (Eurostat 2006) as reference

*New and recurrent cases

^1 ^recognized myocardial infarction

^2 ^unrecognized myocardial infarction

Rates per 100,000 person-years (95% Confidence interval)

## Discussion

This study confirms the magnitude of the impact of MI in the Spanish elderly population, substantially higher than in those younger than 65 years of age[21,22] Elderly men show higher MI incidence and mortality rates than women, particularly at earlier ages (65-74 years old), but these gender differences tend to narrow as they get older. During the last few years, several epidemiological studies have been initiated in Spain to estimate the MI incidence and mortality. However, they [14,15,23] were limited to middle-age subjects and included scarce number of participants from the eldest groups of age. EPICARDIAN study is the first population-based cohort study in the elderly in Spain, with individual follow-up of study participants for a long period of time (10 years of follow-up). A pilot study reporting the study methods in detail and preliminary results in one (Madrid) of the three areas of the main study have been published recently (11). This cohort has similar socio-demographic characteristics and show a similar cardiovascular risk profile than those described in the scarce the scarce studies carried out in ancients in Spain. There is a Spanish registry, the REGICOR-65 plus[24], that provides information on MI rates in the elderly population of Girona, but the methodology to detect cases is different and only MI cases between 1996 and 1997 were registered, and then it may not represent so accurately the real increase in individual risk as the population get older.

In order to compare our results with other studies we adopted previously used and validated international epidemiological procedures and criteria for the identification and classification of MI[25] Nevertheless, almost all epidemiological studies share similar limitations when trying to identify cardiac events. One important limitation is the difficulty to identify unrecognized MI, which has been described to reach a particularly high proportion in the elderly and postmenopausal women[26] However, we tried to minimize this potential underestimation using multiple specific strategies such as reviewing clinical reports. Other particular limitation is the possible underestimation of ischemic events, especially during the third period of follow-up (1998-2004), due to the elevated proportion of not contacted subjects (n = 631; 17%). Nevertheless we used secondary sources of information in order to detect possible cases of MI in all of these subjects. It should be also emphasized that the diagnosis of MI was not only based on the ECG, but also on the clinical symptoms and the results of laboratory testing (CK-MB elevations). One might always speculate that the application of more recent diagnostic tools, such as troponin levels, might have revealed a higher proportion of MI cases, as has been estimated by other studies[27] A possible underestimation of the impact of MI in our population can also be explained because of the exclusion of institutionalized subjects and persons with evidence of relevant disabilities and life threatening diseases.

Once these potential limitations are assumed, the incidence of definite MI seems to be in accordance with other studies carried out in Spain describing higher incidence rates amongst the elderly than rates in middle age-adults. This finding is of crucial importance since elderly people are the fastest growing population segment in western countries and, in consequence, demand for health resources of diagnosis and treatment is bound to increase[28] When comparing our results to those of the REGICOR-65 plus study[24] we observed higher absolute values in our study but both confirm an overall increase with age, and higher risk in men than in women (table 4). Interestingly, in our study, the age-related increase was progressive in women but not in men, and also in accordance with that observed in other studies, in which a concentration of cases occurred in men between 65-74 years in men[29] and over this age in women[15] The question of whether males prematurely develops CHD because a gender related genetic predisposition or whether their occurrence is delayed in women because of a shorter exposure to relevant cardiovascular risk factors arises. The relative higher increase of MI risk with age, observed in elderly females, could be related to the higher burden of cardiovascular risk factors described in the postmenopausal women (see table 1).

In the international context, the incidence rates in our study are lower than other European[26,30] and American [31-33] cohort studies (table 4), but age- and sex- distributions are quite similar. This finding can be explained by a real higher MI incidence in north-European and American elderly populations, as it has been described for middle-age adult population in the International MONICA study. Since Spanish elders, as middle-aged adults, show a high prevalence of cardiovascular risk factors[11], the intriguing paradox of a low MI incidence and mortality in southern Europe despite the high cardiovascular risk factor prevalence[34] is likely to hold in older ages.

Men showed a significantly higher CHD mortality compared with that of women, but gender differences are less evident when specific mortality rates (MI rates instead of CHD rates) are considered. A recently published population-based myocardial infarction (MI) register in persons aged 75 to 99 years describes a CHD annual mortality declination among men by 3.5% and 1.0% in the group 75 to 99-years-old. Among women, it declined by 2.2% per year in the 75- to 84-year-old age group but increased by 1.3% per year in the 85- to 99-year-old age group. MI attack rate did not change in men but increased significantly in women aged 85 to 99 years. [34] Previous research indicates significant age-associated differences in clinical characteristics in elderly patients with AMI, which account for some of the age-associated differences in mortality. Older age is associated with a higher prevalence of co-morbid conditions, atypical AMI presentation, non-diagnostic electrocardiograms, complications and mortality and with significant cardiovascular structural and physiologic changes that might predispose patients to adverse outcomes, including abnormalities of left ventricular diastolic function, decrease in systemic vascular compliance, increased coagulation factors, and altered neurohormonal and autonomic influences. [35]

Lastly, increased fatality with age may be related to lower effectiveness of life-saving treatments, lower hospitalization rates, lower use of diagnostic and therapeutics procedures, longer delay between onset of symptoms and admission to the emergency room and more co-morbidity in the elderly [36-38].

Conclusion

In summary, we conclude that incidence of fatal and non-fatal MI is high in the Spanish elderly population. Overall MI impact remains greater in men compared to women but gender differences tend to narrow as male and female get older. The importance of including older patients in strategies to the prevention and management of MI should be considered

## Competing interests

The authors declare that they have no competing interests.

## Authors' contributions

-RG was the principal investigator and developed the conception and design, participated in the analysis and interpretation of data, in drafting the manuscript, revising it critically for important intellectual content; and have given final approval of the version to be published.

-MA and BR participated in acquisition of data, analysis and interpretation of data, were involved in drafting the manuscript and have given final approval of the version to be published.

-JM, SV, IL, BN, CS and FRS participated in study design, coordination of field studies, analysis and interpretation of data; have been involved in revising the manuscript critically for important intellectual content; and have given final approval of the version to be published. All authors read and approved the final manuscript.

-EPICARDIAN Group: The members of the group conducted the field studies in each geographical area

## Pre-publication history

The pre-publication history for this paper can be accessed here:


